# Unilateral stimulation of the lateral division of the dorsal telencephalon induces synaptic plasticity in the bilateral medial division of zebrafish

**DOI:** 10.1038/s41598-017-08093-9

**Published:** 2017-08-22

**Authors:** Yao-Ju Wu, Yu-Lan Chen, Tso-Hao Tang, Ming-Chong Ng, Tamara G. Amstislavskaya, Maria A. Tikhonova, Yi-Ling Yang, Kwok-Tung Lu

**Affiliations:** 1 Department of Life Science, National Taiwan Normal University, Taipei, Taiwan; 2 Center for General Education, National Quemoy University, Quemoy, Taiwan; 3 Laboratory of Experimental Models of Neurodegenerative Processes, Federal State Budgetary Scientific Institution “Scientific Research Institute of Physiology and Basic Medicine” (SRIPhBM), Novosibirsk, and Novosibirsk State University, Novosibirsk, 630090 Russia; 4 Department of Biochemical Science and Technology, National Chia-Yi University, Chia-Yi, Taiwan

## Abstract

This study was aimed to evaluate the synaptic plasticity in projections from the dorsal lateral region (Dl) to the bilateral dorsal medial region (Dm) of the zebrafish telencephalon. The results showed that unilateral electrical stimulation of the Dl evokes a negative field potential (FP) in both the contralateral and ipsilateral side of the Dm. We tested synaptic plasticity, including high-frequency stimulation-induced LTP (HFS-LTP) and low-frequency stimulation-induced LTD (LFS-LTD). We demonstrated that HFS-induced bilateral LTP is NMDAR-dependent by the application of an NMDAR antagonist, DL-AP5 (30 μM, suprafused for 10 min), which blocked the HFS-induced LTP in both the contralateral and ipsilateral Dm. In addition, LTP was restored after DL-AP5 was washed out by continuous aCSF suprafusion. These results suggested that the potentiation is NMDAR-dependent. Either LFS (1 Hz for 20 min) or applying the mGluR agonist, DHPG (40 μM, suprafused for 10 min) successfully induced bilateral LTD for at least 1 h. Furthermore, both the contralateral fEPSP and LTP vanished after ablation of the anterior commissure. In conclusion, the results of the present study suggested that the projection between the Dl and contralateral Dm in the telencephalon of zebrafish is via the anterior commissure and possesses synaptic plasticity.

## Introduction

Previous studies have indicated that the telencephalon is crucial to neural circuits enabling emotional and spatial learning in teleost fish^1–3^. Neurochemical staining methods and neuroanatomical analyses have divided the teleost telencephalon into two major areas: the dorsal telencephalic area of the pallium and the ventral telencephalic area of the subpallium^4^. Further studies have shown that the dorsal telencephalon of zebrafish can be categorized into three regions along the medial-lateral axis, including the dorsal lateral (Dl), dorsal medial (Dm) and dorsal posterior (Dp). The Dl, Dm and Dp regions of zebrafish have also been proven to be respectively homologous to the hippocampus, amygdala and piriform cortex in mammals^5, 6^.

Folgueira *et al*. used the fluorescent dye DiI to trace the connections within the telencephalon. By applying DiI to the Dl division, the fibers at different rostrocaudal levels were shown to cross the anterior commissure from the Dl to the Dm division of the contralateral side. The exact functional role of those connections has remained uncertain^7, 8^. Our previous results showed that electrical stimulation in the Dl division could evoke a field potential in the ipsilateral Dm division. In addition, long-term potentiation (LTP) and long-term depression (LTD) were respectively induced by applying high-frequency stimulation (HFS) and low-frequency stimulation (LFS)^9, 10^. These observations raise the possibility that the activation of the unilateral Dl division may induce synaptic plasticity on both sides of the Dm division. In this study, we used a multi-electrode dish 64-channel system (MED64) to assess the synaptic plasticity in the Dl-Dm pathway of both the ipsilateral and contralateral sides of the telencephalon.

## Results

### Stimulation of the unilateral side of the Dl evoked a field potential in both the ipsilateral and contralateral sides of the Dm

**Figure 1:**
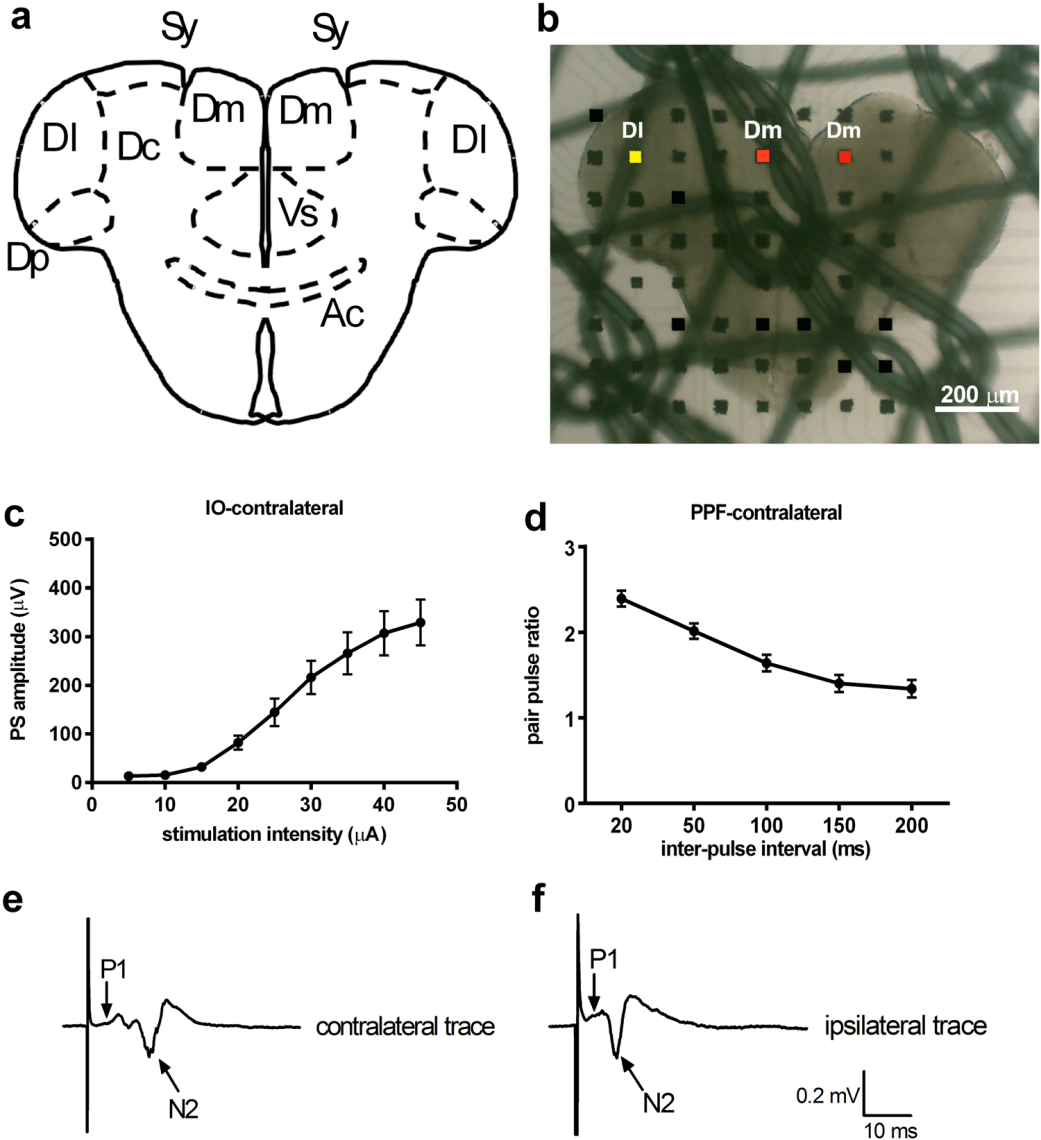
Stimulation of the unilateral side of the Dl evoked a field potential in both the ipsilateral and contralateral side of the Dm. (**a**) Illustration diagram of the zebrafish telencephalon brain slice. Dl, lateral zone of D; Dm, medial zone of D; Dc, central zone of D; Dp, posterior zone of D; Cantv, commissura anterior, pars ventralis; Sy, sulcus ypsiloniformis; Vs, supracommisural nucleus of ventral telencephalic area. The diagram and the area are referred from Wullimann^32^. (**b**) The brain slice was placed over the electrodes on the MED probe, and a stimulating cathode (yellow square) and two recording cathodes (red square) were chosen. (**c**) The input-output relationship of the Dl-Dm pathway of the contralateral side. (**d**) Paired-pulse facilitation of the Dl-Dm pathway of the contralateral side. (**e**) Single trace of the components of the contralateral Dm field potential. (**f**) Single trace of the components of the ipsilateral Dm field potential.

For electrophysiological recordings, 350-μm-thick brain slices were placed on the recording MED probe (Fig. 1b). Bipolar stimulation was applied to the Dl division by using the posterior (Dp) zones and sulcus ypsiloniformis (Sy) as landmarks (Fig. 1a,b). Signals were recorded in both the ipsilateral and contralateral Dm division (Fig. 1e,f). We characterized the neuronal properties by the input-output response, such as the input-output (IO) curve and pair-pulse facilitation (PPF). Stimulating the Dl division produced a population spike (PS) in the Dm division of the contralateral side. As shown in Fig. 1c, the amplitude of the PS in the Dm division of the contralateral side gradually increased with the stimulation amplitude from the threshold value to a smoothly saturated form. In Fig. 1d, the Dm division of the contralateral side showed paired-pulse facilitation, which indicated that the unilateral stimulus induced a bilateral response in the contralateral Dm (PPF, n = 6. IO, n = 6, both ipsilateral and contralateral).

### High-frequency stimulation of the unilateral side of the Dl evoked long-term potentiation on both the ipsilateral and contralateral sides of the Dm

**Figure 2:**
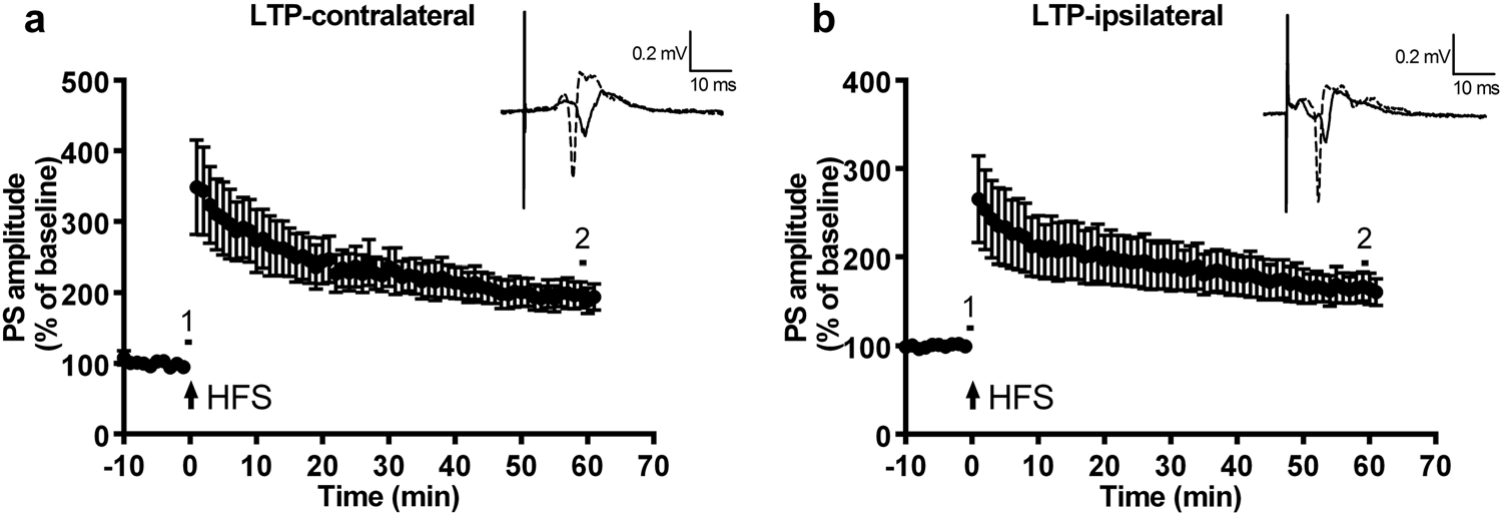
High-frequency stimulation of the unilateral side of the Dl evoked long-term potentiation (LTP) in the ipsilateral and contralateral side of the Dm. (**a**) LTP of the contralateral side of the Dm (group data). (**b**) LTP of the ipsilateral side of the Dm (group data). High-frequency stimulation (100 Hz) was applied (arrow) in the Dl division. The amplitude of the PS of both sides after LTP induction was 1.5 times larger than the amplitude at baseline, and this increase could last at least 1 hour. Using paired t-test to compare the amplitude at 60 min (point 2) with the amplitude at baseline (point 1): (193.89 ± 17.31%, n = 8, p = 0.0010; ipsilateral: 164.83 ± 16.05%, n = 8, p = 0.0012). Each point represents the mean ± SEM of the PS amplitude. n = 8.

The results are shown in Fig. 2. Following HFS given in the Dl division, LTP can be induced in the Dm division of the ipsilateral and contralateral sides. The amplitude of the PS of both sides after LTP induction was 1.5 times larger than the baseline amplitude, and this increase could last over 1 hour. The PS amplitude of the contralateral Dm and ipsilateral Dm at 1 hour after HFS were 193.89 ± 17.31% (mean ± S.E.M., n = 8, p = 0.0010) and 164.83 ± 16.05% (n = 8, p = 0.0012), respectively. We demonstrated that applying HFS to one side of the Dl could induce LTP in the Dm division of both the ipsilateral and contralateral sides. In addition, the latency of the initial positive deflection of the contralateral Dm lasted longer than that of the ipsilateral Dm.

### NMDA receptor antagonist, DL-AP5, blocked the formation of LTP in both Dl-Dm pathways of the contralateral and ipsilateral sides

**Figure 3:**
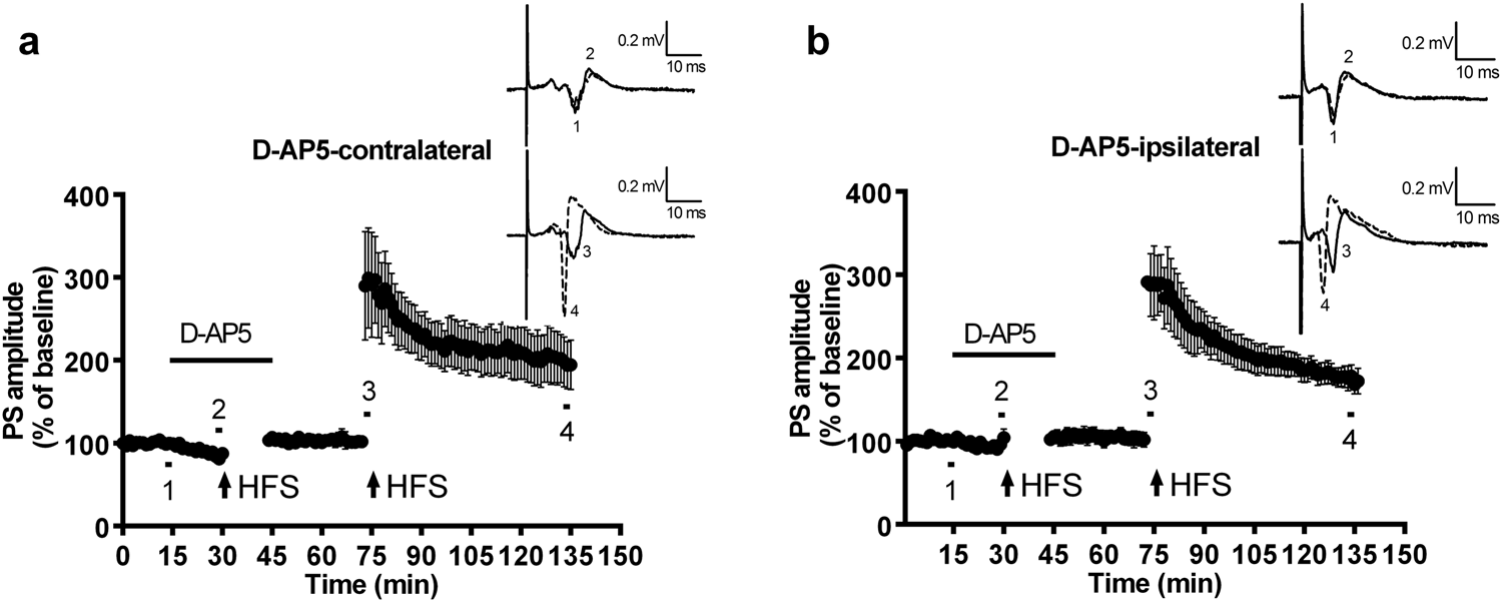
NMDA receptor antagonist, D-AP5, blocked the formation of LTP in the Dl-Dm pathways of both the contralateral and ipsilateral side. (**a**) Effect of D-AP5 on LTP induction in the contralateral side with Dm induction (group data). (**b**) Effect of D-AP5 on LTP induction in the ipsilateral side with Dm induction (group data). Here, 30 µM of D-AP5 (solid line) was applied after 10 min of baseline recording. The first HFS (arrow) was delivered 15 min after the start of drug perfusion (point 2 compared to point 1, contralateral: 87.57 ± 3.97%, n = 9, p = 0.0576; ipsilateral: 104.3 ± 9.93%, n = 9, p = 0.9529). The second HFS (arrow) was delivered 30 min after the washout of D-AP5. LTP was not induced under the effect of D-AP5 (point 3 compared to point 1, contralateral: 100.8 ± 7.71%, p = 0.9977; ipsilateral: 100.5 ± 7.59%, p = 0.999), but the second HFS was able to induce LTP after D-AP5 washout, which lasted at least 1 hour (point 4 compared to point 1, contralateral: 194.5 ± 28.97%, p = 0.0323; ipsilateral: 168.2 ± 11.78%, p = 0.0012). Values for each side were tested by using one-way ANOVA. Post hoc analyses were conducted using Dunnett’s test. Each point represents the mean ± SEM of the PS amplitude. n = 9.

The results are summarized in Fig. 3. The PS amplitude of the contralateral Dm and ipsilateral Dm 15 min following application of D-AP5 were 87.57 ± 3.97% (n = 9, p = 0.0576) and 104.3 ± 9.93% (n = 9, p = 0.9529), respectively, suggesting that application of 30 μM of D-AP5 alone did not significantly change the field potential. During the application of D-AP5, the Dm divisions of the ipsilateral and contralateral sides failed to undergo LTP. The PS amplitude of the contralateral Dm 30 min following the first HFS was 100.8 ± 7.71% (p = 0.9977); the PS amplitude of the ipsilateral Dm 30 min following the first HFS was 100.5 ± 7.59% (p = 0.999). LTP was restored after washout of DL-AP5. The PS amplitudes of the contralateral and ipsilateral Dm 10 min following the second HFS were 224.8 ± 28.12% (p = 0.0026) and 240.7 ± 33.36% (p = 0.0086), respectively. Sixty minutes following the second HFS, the PS amplitudes of the contralateral and ipsilateral Dm were 194.5 ± 28.97% (p = 0.0323) and 168.2 ± 11.78% (p = 0.0012), respectively. These results suggested that HFS-induced LTP requires the activation of NMDA receptors in the contralateral Dm.

### Low-frequency stimulation or mGluR-1 agonist administration evoked long-term depression (LTD) in the ipsilateral and contralateral sides of the Dm

**Figure 4:**
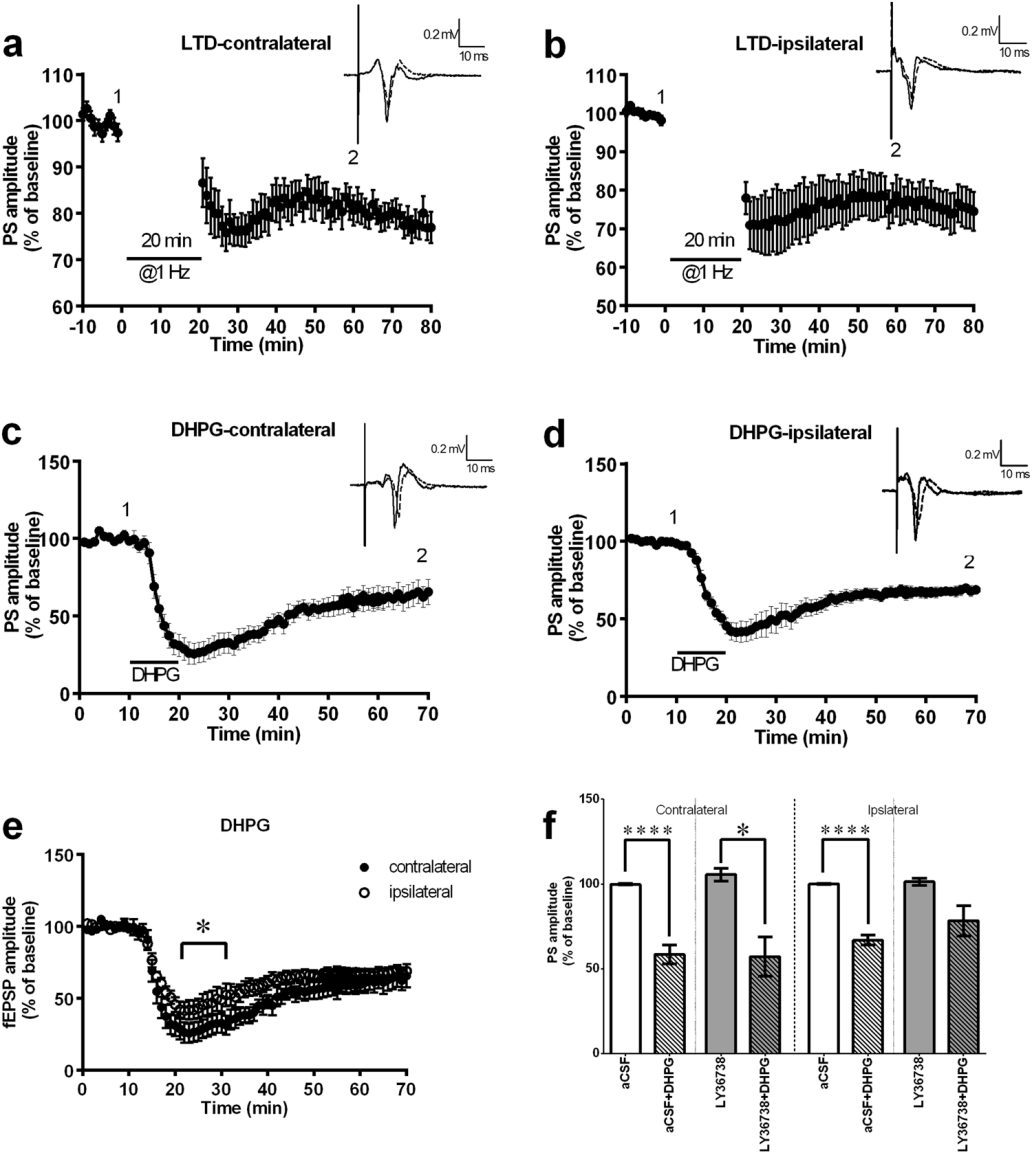
Low-frequency stimulation and DHPG evoked long-term depression (LTD) in the ipsilateral and contralateral side of the Dm. (**a**) LTD of the contralateral side of the Dm. (**b**) LTD of the ipsilateral side of the Dm. Low-frequency stimulation (1 Hz) was applied (arrow) in the Dl division. The amplitude of the PS in both sides after LTD induction was 80% smaller than the baseline amplitude, and this reduction could last at least 1 hour (point 2 compared to point 1, contralateral: 77.13 ± 3.34%, n = 11, p = 0.0988; ipsilateral: 75.92 ± 6.11%, n = 11, p = 0.1525). Each point represents the mean ± SEM of the PS amplitude. n = 11. (**c**) The effect of DHPG on LTD induction in the contralateral side with Dm induction. (**d**) The effect DHPG on LTD induction in the ipsilateral side with Dm induction. The amplitude of the PS in both sides after LTD induction was 70% smaller than the baseline amplitude, and this reduction could last at least 1 hour (point 2 compared to point 1, contralateral: 62.42 ± 7%, n = 9, p = 0.0001; ipsilateral: 67.17 ± 3.1%, n = 9, p < 0.0001). (**e**) The comparison of the PS amplitude with DHPG-induced LTD in the contralateral and ipsilateral Dm. The PS amplitude of the contralateral side was smaller than that of the ipsilateral side in the 25–35-min period (n = 9, F_(1, 16)_ = 4.809, p = 0.0434). DHPG (40 µM) was applied for 10 min after 10 min of baseline recording. (**f**) Co-administration of the mGluR antagonist LY36738 significantly attenuated the DHPG-induced contralateral LTD but not the ipsilateral LTD. (n = 5, p = 0.0134 & p = 0.0536 compared with corresponding control group respectively) Each point represents the mean ± SEM of the PS amplitude. n = 9.

Results are shown in Fig. 4. Following LFS given in the Dl division, LTD can be induced in the Dm division of the ipsilateral and contralateral sides. The PS amplitude after LTD induction in both sides was 80% smaller than the baseline amplitude, and this reduction in amplitude could last at least 1 hour. The PS amplitude of the contralateral Dm 1 hour following LFS was 77.13 ± 3.34% (n = 11, p = 0.0988) (Fig. 4a); the PS amplitude of the ipsilateral Dm 1 hour following LFS was 75.92 ± 6.11% (n = 11, p = 0.1525) (Fig. 4b). From the results, we can infer that applying LFS to one side can induce synaptic plasticity in the form of LTD in the Dm division of both the ipsilateral and contralateral sides. Previous results suggested that the metabotropic glutamate receptor (mGluR) was involved in LTD formation in the Dl of zebrafish^9, 10^. We then tested whether suprafusion of the mGluR1 and mGluR5 agonist DHPG caused LTD in both the ipsilateral and contralateral Dm division (Fig. 4c,d). The PS amplitude on both sides was 70% smaller than the baseline and lasted more than 1 hour. The PS amplitude of the contralateral Dm 1 hour following application of DHPG was 62.42 ± 7% (n = 9, p = 0.0001); the PS amplitude of the ipsilateral Dm 1 hour following application of DHPG was 67.17 ± 3.1% (n = 9, p < 0.0001). The results suggested that the application of DHPG induced synaptic plasticity in the form of LTD in the Dm division of both the ipsilateral and contralateral sides. In Fig. 4e, significant differences are found in the PS amplitude after DHPG-induced LTD in the contralateral and ipsilateral Dm during the early phase of LTD (25–35 min). The PS amplitude of the contralateral side was smaller than that of the ipsilateral side in the 25–35-min period (n = 9, F_(1, 16)_ = 4.809, p = 0.0434) (Fig. 4e). In addition, the mGluR1 antagonist LY36738 was used for verification. The results showed that neither the contralateral nor ipsilateral LTD was blocked by LY36738 (n = 5, p = 0.0134 & p = 0.0536 compared with the corresponding control group, respectively) (Fig. 4f).

### Both the contralateral fEPSP and HFS-LTP vanished after anterior commissure ablation

**Figure 5:**
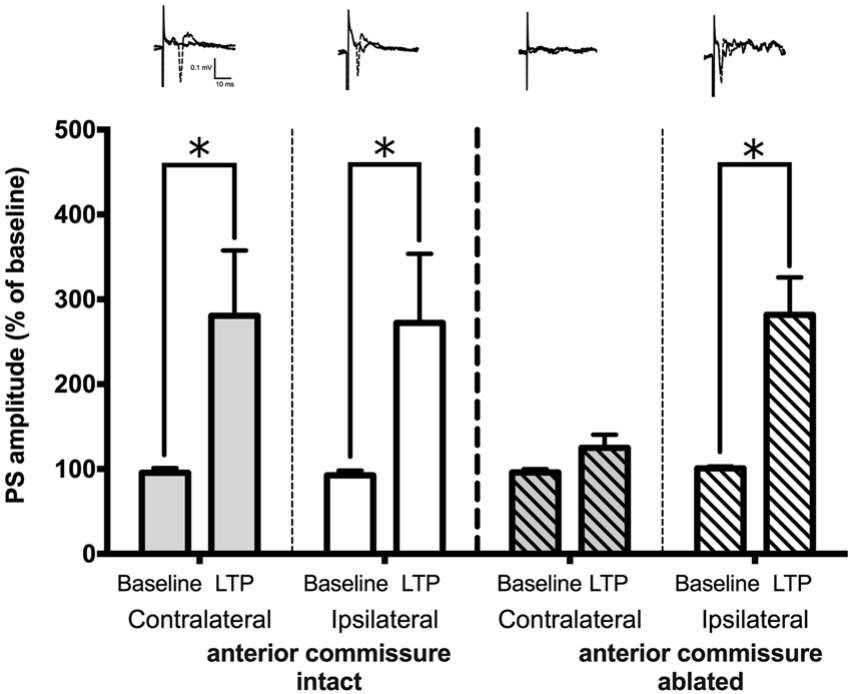
Both the contralateral fEPSP and HFS-LTP vanished after anterior commissure ablation. Without the anterior commissure being severed, both the ipsilateral and contralateral HFS-LTP remained intact (p = 0.0223 and 0.0194 respectively, intact). When the anterior commissure was ablated before recording, both the contralateral fEPSP and HFS-LTP vanished without any impairment in the ipsilateral HFS-LTP (Ipsilateral and contralateral, p = 0.0403 & 0.714 respectively, ablated). Each point represents the mean ± SEM of the PS amplitude.

The identity of the axon pathway that connects the Dl to the contralateral Dm is unclear. Previous tracing studies have suggested that neurons in the pallium project to and synapse on cells of the sub-pallium. Unlike mammals, there is no corpus callosum in zebrafish. Therefore, the anterior commissure is the most likely candidate. To test this hypothesis, we used a microfilament to ablate the anterior commissure of the zebrafish brain slice before placing the slice into the recording chamber. The results showed that without the anterior commissure ablation, both the ipsilateral and contralateral HFS-LTP remained intact (p = 0.0223 and 0.0194 respectively, Fig. 5 intact). Both the contralateral Dm fEPSP and HFS-LTP vanished after anterior commissure ablation (Ipsilateral and contralateral, p = 0.0403 & 0.714 respectively)(Fig. 5, ablated). This result suggested that the projection between the Dl and contralateral Dm in the telencephalon of the zebrafish is found in the anterior commissure and possesses synaptic plasticity.

## Discussion

The present study used electrophysiological approaches and a zebrafish model to characterize the synaptic plasticity of the contralateral dorsal lateral (Dl) and dorsal medial (Dm) pathways within the dorsal telencephalon. We found that the latency of the initial positive deflection of the contralateral side lasted longer than that of the ipsilateral side, which might be due to the different distances the stimuli must travel through the neural projection to the recording cathode between the contralateral and ipsilateral sides. A previous study showed the field potential is composed of non-synaptic (P1) and synaptic (N2) components according to the variance in the potential amplitude in the intensity-response curve (IO-curve), paired-pulse facilitation and application of tetrodotoxin (TTX), a strong inhibitor of voltage-gated sodium channels^9^. The P1 component remained static in paired-pulse facilitation and varied in the IO-curve. These results correspond to the observation that a positive deflection fiber volley (FV) waveform was evoked in the CA3 division following electrical stimulation of hippocampal mossy fibers^11^; therefore, the P1 component was identified as a fiber volley. In the present study, the field potentials recorded in the Dm of the contralateral and ipsilateral sides both showed that the Pl amplitudes of both sides increased along with the increase in the stimulus intensity in the IO-curve, while the Pl amplitude of both sides was identical following paired-pulse stimulation. These results reflect those of Ng *et al*. in which the P1 component was shown to be non-synaptic and the N2 synaptic^9^. This point can be further elaborated by applying Ca^2+^ and Mg^2+^ solution to assess the ionic channels underlying the field potential transduction. Which structure connects the Dl to the contralateral Dm is unclear. Previous tracing studies have suggested that the anterior commissure is a possible candidate, particularly because no corpus callosum is found in zebrafish. We also tested this hypothesis by severing the anterior commissure. The results showed that both contralateral LTP and LTD were blocked and ipsilateral LTP and LTD remained intact (Fig. 5), proving that axons traveling through the anterior commissure mediate these processes.

The present study extends our previous observations showing that electrical stimulation of the Dl division can provoke both LTP and LTD in the ipsilateral Dm division. Our previous results also demonstrated that glutamatergic neurotransmission is involved in the formation of both LTP and LTD. Co-administration of the NMDA receptor agonist AP5 blocked the formation of HFS-induced LTP in the Dm division. Either LFS or suprafusion of the metabotropic glutamate receptor agonist DHPG induced LTD in the Dm division^9, 10^.

Our results showed that a stable, robust LTP in the Dm divisions of both the ipsilateral and contralateral sides can be induced by stimulation from either the left or right Dl. Similar results of LTP induction in the contralateral hippocampus of other species have been observed, including in guinea pigs^12^ and mice^13^, implying a high degree of evolutionary conservation in the neural mechanism of neuroplasticity among different species. Most previous studies of neuronal transduction between the two hemispheres have used *in vivo* experiments due to limitations related to brain slice preparation. The complex structure and neuronal circuit of mammalian brains might be more vulnerable during the brain slice preparation process due to the larger brain size.

Several studies have indicated that LTP induction is NMDA-dependent in the CA1-CA3 connection of the hippocampus^14, 15^ and zebrafish telencephalon^9, 16^, which is consistent with our previous data that application of the NMDA receptor antagonist D-AP5 blocked ipsilateral LTP formation, which can be restored after D-AP5 washout^9^. Similar results are also found for LTP induction from the Dl division to the contralateral Dm division in the telencephalon of zebrafish. This suggests that zebrafish can be used as an alternative model for studying NMDA-dependent neuroplasticity in vertebrates.

The Dl and Dm of zebrafish are highly homologous to the hippocampus, amygdala and piriform cortex in mammals. Activation of NMDA receptors is widely accepted to trigger LTP in hippocampal slices, is related to the cellular mechanism responsible for synaptic plasticity and results in learning and the formation of memory in rodents^17–20^. In goldfish and zebrafish, NMDARs are essential for both avoidance learning^21^ and spatial learning^22^. Interestingly, several studies have indicated that the allocation of NMDA receptor subunits is asymmetrical in hippocampal CA1-CA3 circuitry, which may produce unequal numbers of NMDA receptors, thus resulting in a distinct ability to express synaptic plasticity^23, 24^. This may account for the functional dominance of different hemispheres. We speculate that similar phenomena may also occur in the Dl-Dm circuitry of zebrafish telencephalon. This hypothesis could be tested through subsequent experiments such as by comparing the difference between contralateral LTP induced by the left and right Dl and using immunohistochemistry and/or real-time PCR to determine the possible difference in NMDA expression between both sides.

In addition to bilateral LTP formation, we also demonstrated that either LFS or suprafusion of the mGluR agonist DHPG could also trigger a stable, robust LTD in the Dm divisions of both the ipsilateral and contralateral sides, which was consistent with previous results of DHPG-induced LTD in the unilateral hippocampal CA1 region^25–27^. We co-administered LY36738, an mGluR1 antagonist, to further investigate this LTD. The results showed that LY36738 failed to completely block the DHPG-induced ipsilateral LTD (n = 5, p = 0.0134) and contralateral LTD (n = 5, p = 0.0536) (Fig. 4f), implying that the contralateral LTD is largely driven by mGluR5. In the present study, only a single mGluR1 antagonist was applied; we cannot exclude the possibility that there could be some contribution from mGluR1 and possibly NMDA receptors^28^. Future studies could help to clarify the mechanism of LTD induction in the different hemispheres through administration of a specific mGluR5 antagonist or separation of stimuli into different sides of the Dl. mGluRs are well known to regulate the activity of neuronal ion channels, such as AMPA receptors. A previous study indicated that DHPG-induced LTD was related to the internalization of AMPA receptors, removing AMPA receptors from the membrane^29^. There are two major types of LTD including N-methyl-D-aspartate receptor dependent LTD (which is reversible and independent of protein synthesis in the early phase) and metabotropic glutamate receptor dependent LTD (which is triggered by the activation of group I metabotropic glutamate receptors and requires new protein synthesis). Recently, Zurawek and colleagues have proven that the expression of mGluR5 can be regulated by ketamine, an NMDA receptor antagonist. The possible involvement of NMDA receptors deserves further investigation. Based on our results, we speculate that the molecular mechanism that regulates LTD formation is similar for both adult zebrafish and mammals. Further experiments, such as a comparison of the AMPA receptor expression among the bilateral Dm divisions, will be helpful for testing our hypothesis.

In conclusion, the results of the present study suggested that the projection between the Dl and contralateral Dm in the telencephalon of zebrafish possesses synaptic plasticity. The zebrafish model not only provides advantages in genetic and developmental studies^30^ but also provides a model to evaluate cerebral lateralization through electrophysiological research.

## Methods

### Zebrafish

Zebrafish (AB strain) were housed in the National Taiwan Normal University (NTNU) animal care facility on a 14-h light: 10-h dark cycle. The zebrafish were fed twice daily, and the temperature of the water was fixed at 26–28 °C. Spawn were kept in an 8 × 8-cm plastic box using breeding methods from The Zebrafish Book^31^. All procedures were approved and supervised by the NTNU institutional animal care and utilization committee (IACUC) and all experiments were performed in accordance with relevant guidelines and regulations.

### Zebrafish brain slice preparation

Adult zebrafish were sacrificed by euthanasia in an artificial cerebrospinal fluid (aCSF) solution at 0~4 °C containing the following ingredients (in mM): 117 NaCl, 4.7 KCl, 1.2 NaH_2_PO_4_, 2.5 NaHCO_3_, 1.2 MgCl_2_, 2.5 CaCl_2_, and 11 d-( + )-glucose. Zebrafish brains were rapidly placed in the aCSF solution and immersed in a 4% low-melting-point agarose (MDBio, Inc., Taiwan). Transverse telencephalic slices (350 μm) were made by using a vibratome (MA752, Campden Instruments Ltd., UK). Brain slices were then incubated in oxygenated (95% O_2_/5% CO_2_) aCSF solution to stabilize for at least 1 hour prior to recording^9, 10^.

### Electrophysiological recording

A multi-electrode dish 64-channel system (MED64, Alpha MED Sciences, Tokyo, Japan) was used to record extracellular PSs. A MED-P515A probe (Alpha MED Sciences, Tokyo, Japan) was applied with 64 microelectrodes (50 μm × 50 μm) arranged in an 8 × 8 grid with an inter-electrode spacing of 150 μm. To increase cellular adhesion, the MED64 probe was pretreated with 0.1% polyethyleneimine (Sigma, St. Louis, MO, USA) dissolved in 25 mM borate buffer (pH 8.4) at room temperature overnight before use. Before electrophysiological recordings, a brain slice was carefully moved into the recording MED64 probe and perfused with aCSF (28 °C) using a peristaltic pump (Gilson Minipuls 3, Villiers Le Bel, France). One of the 64 microelectrodes was chosen as a stimulating cathode in the Dl division, and the remaining 63 microelectrodes served as recording cathodes. Biphasic rectangular current pulses (0.2 ms duration) were applied every 20 s, and the recording cathode was placed at the contralateral side of the Dm. In this study, the maximum PS response was defined by increasing the stimulus intensity until an asymptotic limit was reached. The current that triggered a response approximately 30 to 50% of that of the maximal response was used throughout the experiment. Generally, basal synaptic transmission measurements included input-output (I/O) functions and short-term plasticity (paired-pulse facilitation, PPF). I/O curves were obtained from 9 incremental stimulation intensities. The inter-pulse intervals were 20, 50, 100, 150, and 200 ms for the paired-pulse stimulation. After a stable baseline recording, LTP was elicited by HFS protocols consisting of three stimulus trains of 100 pulses (at 100 Hz) with 20-s inter-train intervals. LTD was induced by low-frequency stimulation that consisted of 1-Hz stimulation for 20 min. The magnitudes of both LTP and LTD were measured, post-induction, as an average of 10 min at the end of the recording period^9, 10^.

### Drug preparation and administration

Drugs were freshly prepared from stock solutions and diluted in aCSF. Brain slices were then suprafused with aCSF. 2-amino-5-phosphopentanoate (DL-AP5) and 3,5-dihydroxyphenylglycine (DHPG) were both purchased from Abcam (UK) and prepared in distilled water. Here, 30 µM DL-AP5 was freshly prepared from 1 mM stock solution, and 40 µM DHPG and 80 µM LY367385 were also prepared from 1 mM stock solution.

### Statistical analysis

Values are expressed as the mean ± standard error mean (SEM). The results of the D-AP5 experiment were tested using one-way ANOVA. *Post hoc* analyses were conducted using Dunnett’s test or Fisher’s LSD (anterior commissure ablation experiment). The rest of the data were tested using paired t-test, and p < 0.05 was considered significant. GraphPad PRISM^®^ software version 6.0 was used for statistical analysis.
